# The Future of Cardiac Care: A Review on the Role for Non-Invasive, Home-Based Monitoring and Rehabilitation after Hospitalisation for Acute Decompensated Heart Failure

**DOI:** 10.1007/s11897-026-00778-5

**Published:** 2026-10-09

**Authors:** Niels A. Stens, Yoram Müller-Jabusch, Kiki E. L. A. Veenhof, Wilhelmina F. Goevaerts, Mark Larooi, Jill Bay, Daan M. Janssen, Annelieke Driessen, Isabella Kardys, Hendrik Koffijberg, Frank R. Halfwerk, Robert M. A. van der Boon, Karin Dijkstra, Rudolf B. Kool, Thijs M. H. Eijsvogels, Annemien E. van den Bosch, Hareld M. C. Kemps, Dick H. J. Thijssen

**Affiliations:** 1 Department of Medical BioSciences (928), Radboud University Medical Center, P.O. Box 9101, Nijmegen, 6500 HB the Netherlands; 2 Department of Industrial Design, Eindhoven University of Technology, Eindhoven, The Netherlands; 3 Department of Cardiology, Cardiovascular Institute, Thorax Center, Erasmus MC, Rotterdam, The Netherlands; 4 Department of Cardiology, Maxima Medical Center, Veldhoven, the Netherlands; 5 Research Group Smart Health, School of Health, Saxion University of Applied Sciences, Deventer, The Netherlands; 6 Health Technology and Services Research Department, Technical Medical Center, University of Twente, Enschede, The Netherlands; 7 Thoraxcentrum Twente, Medisch Spectrum Twente, Enschede, The Netherlands; 8 Department of Biomechanical Engineering, University of Twente, Enschede, The Netherlands; 9 IQ Health Science Department, Radboud University Medical Center, Nijmegen, the Netherlands; 10 European Reference Network for Rare, Low Prevalence and Complex Diseases of the Heart (ERN-GUARD-HEART), Amsterdam, The Netherlands; 11 Research Institute for Sport and Exercise Sciences, Liverpool John Moores University, Liverpool, UK

**Keywords:** Heart failure, Remote patient monitoring, Cardiac rehabilitation, Telemedicine

## Abstract

**Purpose of Review:**

Heart failure (HF) is characterised by recurrent decompensation leading to worsening of symptoms and unplanned hospitalisations. Patients often experience poor post-hospitalisation recovery, fail to maintain functional capacity, and demonstrate a high risk for re-admission. In this review we discuss these challenges, and explore how non-invasive, digital solutions may improve health outcomes.

**Recent Findings:**

In contrast to current episodic surveillance, advances in wearable sensor technologies enable continuous, non-invasive monitoring to facilitate physiology-guided management to improve early clinical stabilisation. Additionally, trials have highlighted the potential of exercise-based cardiac rehabilitation to improve functional capacity in HF patients. Finally, continuous monitoring of relevant physiological signals, through developing algorithms, have the potential for early prediction of clinical deterioration.

**Summary:**

Non-invasive, co-created digital solutions may facilitate early stabilisation, improve functional capacity and predict clinical deterioration following episodes of decompensation. Collectively, they may enhance clinical outcomes and quality of life in patients with HF.

## Introduction

Heart failure (HF) is rapidly growing into a major burden for both patients and health systems across the globe [1, 2]. HF confers a 5-year survival of ~ 60% in European countries [3, 4], with even poorer outcomes reported in lower- and middle-income countries [5, 6]. Accordingly, HF markedly reduces quality of life. An important challenge in the treatment of HF is management of acute decompensated heart failure (ADHF), which relates to the (sudden) worsening of clinical symptoms due to pulmonary and systemic congestion, often leading to hospitalisation. Following ADHF hospitalisation, HF patients typically show poor recovery and inability to restore functional capacity. This early post-discharge phase is often referred to as a ‘vulnerable phase’, characterised by a high risk of rehospitalisation [7, 8]. Guideline-directed medical therapy (GDMT) improves survival and reduces hospitalisation risk, but initiation and up-titration are frequently delayed or incomplete following ADHF [9]. Infrequent and one-size-fits-all surveillance during this phase may lead to suboptimal management and delayed treatment adjustments. Indeed, the risk of readmission is increased, with 30-day and 1-year readmission rates reported of 13% and 36%, respectively [7]. Consequently, readmission leads to poor quality of life for patients and their relatives, and is associated with substantial burden on healthcare and society [10–12]. This highlights the importance to improve care following decompensation in patients with HF.

**Fig. 1:**
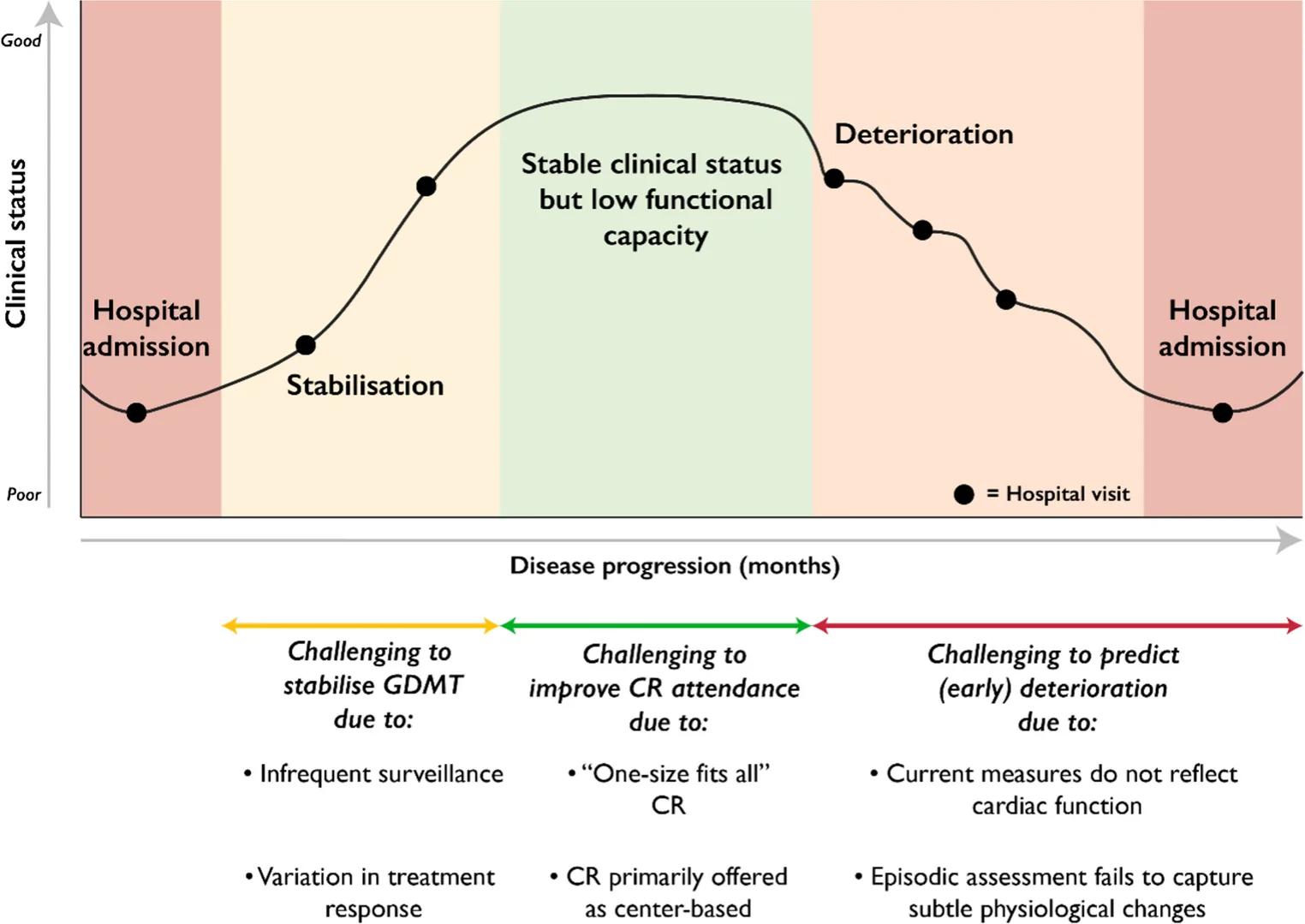
Heart failure disease progression, with contemporary challenges of HF therapy. *Following hospital admission*,* patients frequently still exhibit persistent congestion*,* neurohumoral activation and incomplete hemodynamic recovery. During this vulnerable period*,* rapid but safe GDMT optimisation should be initiated in an attempt to stabilize the patient*,* but is challenging in the context of infrequent visits and by considerable within- and between-patient variation in treatment responses. Following GDMT up-titration*,* patients become increasingly more stable*,* but generally have a low functional status. CR has shown to be effective in improving functional status and clinical outcomes*,* but are frequently prescribed using a “one-size fits all” and center-based approach. Deterioration of clinical status is frequently challenging to predict using traditional measures such as weight management*,* as these not reflect cardiac function. Additionally*,* infrequent and one-size-fits-all surveillance in the post-discharge phase may lead to suboptimal management and delayed treatment adjustments*,* and may result in readmission for ADHF. ADHF acute decompensated heart failure*,* CR cardiac rehabilitation*,* GDMT guideline-directed medical therapy*

Providing care following ADHF is associated with important challenges (Fig. 1). First, heterogeneity in clinical presentation and treatment responses, combined with infrequent hospital visits, makes it challenging to timely adjust therapy to clinical fluctuations. These phenotypic differences may substantially influence treatment response, risk of decompensation, and recovery trajectories [13–15]. Whilst studies on (home-based) monitoring of weight, blood pressure and clinical status in HF demonstrate its feasibility, these parameters poorly reflect cardiac function and are crude measures to fine-tune titration [16] or for early detection of ADHF [17]. This highlights the need for novel approaches to guide therapy.

A second challenge relates to the low levels of functional capacity [18], which may further deteriorate following hospitalisation [19]. Cardiac rehabilitation is an effective intervention for patients with HF to improve functional capacity and subsequently reduce risks for hospitalisation and improve quality of life [20]. Nonetheless, uptake of CR in patients with HF is low, with only 5–10% of patients enrolling in such programs [21, 22], especially in those following ADHF. Uptake of CR is subject to wide global variation, with poorer access to CR in low- and middle income countries [23]. Barriers include frailty, transportation difficulties, low health literacy, socioeconomic disparities, limited referral pathways, and competing comorbidities. These behavioural and system-level factors highlight the need for more accessible and flexible rehabilitation models, including home-based and digitally supported CR programs tailored to individual patients’ needs.

A third challenge relates to the early prediction of deterioration and, hence, the ability to timely adjust pharmacotherapy, particularly to restore fluid balance. Studies have found that days prior to clinical deterioration physiological compensatory adjustments take place in blood volume, leading to increased cardiac filling pressures and, eventually, fluid accumulation [24]. Recent work highlighted the potential for remote invasive monitoring of filling pressures to reduce risk for hospitalisation in patients with HF [25]. We will discuss the current technological developments to non-invasively monitor physiological signals relevant for cardiac function (e.g., filling pressure, heart rate variability, vibrations related to cardiac mechanics, and hemodynamics), and how they form the basis for developing novel algorithms for early prediction of clinical deterioration.

Previous reviews and meta-analyses have generally examined the isolated effects of non-invasive remote monitoring [26, 27] or cardiac rehabilitation [28, 29] following acute decompensation. The underlying mechanisms of these interventions are likely distinct, with remove monitoring improving GDMT and cardiac rehabilitation improving functional capacity. Therefore, combining both may have synergistic effect, further reducing hospitalisation risk. However, no review has brought these evidence bases together, nor have considered potential interplay between these fields.

Taken together, we discuss three key challenges that contribute to high re-admission rates following acute decompensated HF: (1) Poor stabilisation due to within/between-subject variation in treatment response, (2) Low functional capacity, and (3) Limited ability to timely predict clinical deterioration. We outline potential solutions, including home-based wearables for personalized, continuous monitoring and treatment optimization, home-based cardiac rehabilitation to improve functional capacity, and data-driven innovations for early prediction of clinical deterioration. Finally, we discuss pathways towards clinical implementation, where we emphasize the role of actively engaging stakeholders (e.g. patients, caregivers, healthcare professionals, and policymakers) in co-creating context-sensitive, effective and sustainable real-world innovations. In addition, we will also discuss the role of health technology assessment, which is becoming increasingly important for clinical translation and implementation given the rapid (technological) innovation combined with the high financial burden and limited staffing capacity within healthcare systems.

## Poor Stabilisation after HF Hospitalisations: Monitoring to Anticipate within/between-subject Variation

The early weeks following hospital discharge for ADHF represent a critical stabilisation phase, and is focused on up-titration of GDMT [30]. A key challenge during this period is that patients frequently exhibit persistent congestion, ongoing neurohormonal activation, and incomplete haemodynamic recovery [31]. This makes frequent monitoring of the individual patient important to adjust (pharmacological) treatment to optimize the patient’s situation. Traditional monitoring of these symptoms relies on episodic measurements of vital signs (e.g., heart rate and blood pressure), body weight, and self-reported symptoms [32, 33]. Since these parameters are relatively insensitive to early, rapid haemodynamic changes [9, 32], up-titration of GDMT to clinical fluctuations is challenging. This observation is further complicated by the strong variation in treatment response between patients, as well as the thin line between the benefits of rapid GDMT optimisation and the potential risks (e.g. hypotension, renal dysfunction, and/or recurrent decompensation). As a consequence, physicians seem hesitant to up-titrate GDMT due to safety concerns between episodic visits [34]. Since congestion may develop within days to weeks before onset of symptomatic deteriorations [35], widening the therapeutic window for earlier intervention, through more frequent monitoring of vital signs, is a key priority to improve outcomes following acute decompensation.

**Fig. 2:**
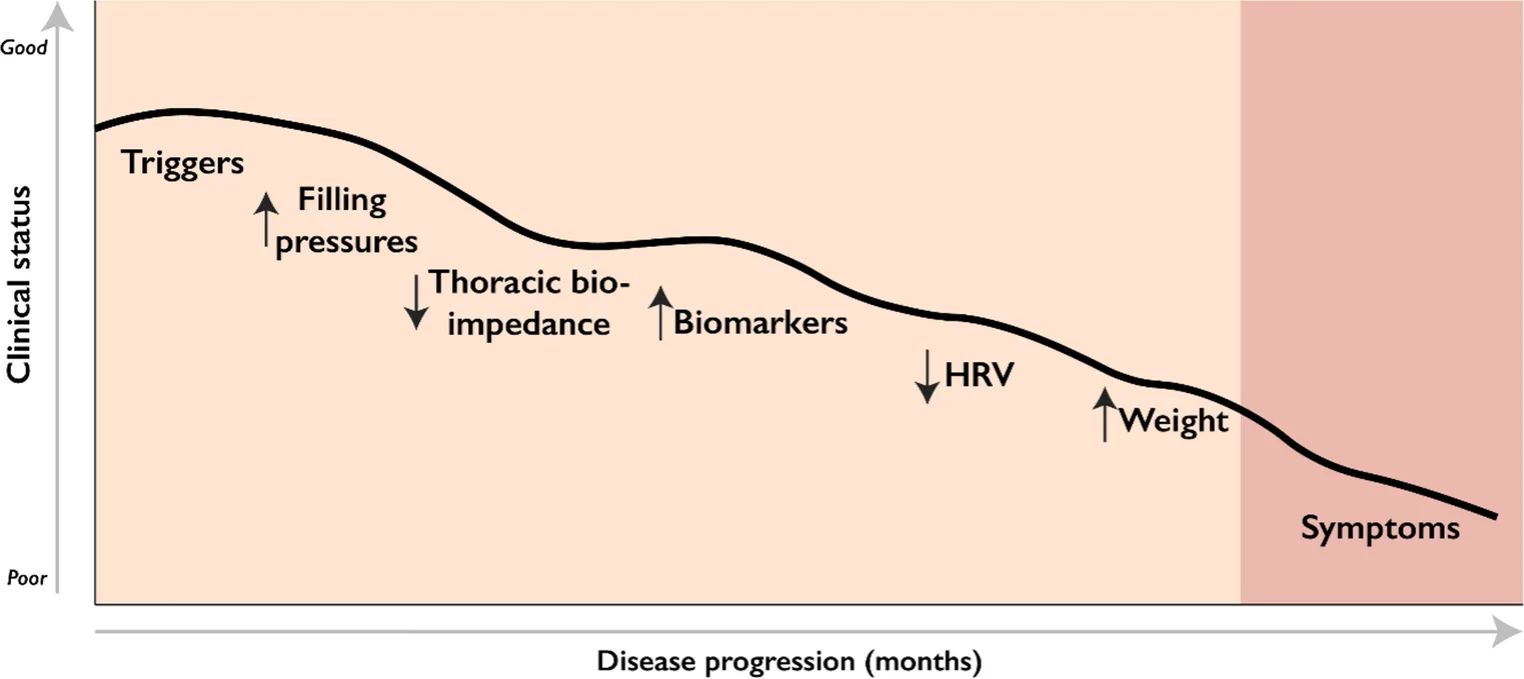
Heart failure disease progression with changes in physiological parameters. *Distinct changes in physiological parameters precede both weight gain and the onset of symptoms. HRV heart rate variability*

A potential solution to better inform up-titration of GDMT includes the adoption of physiology-guided devices. In contrast to conventional measurements, continuous monitoring of relevant haemodynamic surrogates are sensitive to changes before onset of symptoms [36]. For example, rises in cardiac filling pressures and plasma volume expansion are frequently observed before weight gain and onset of dyspnoea (Fig. 2). Recent work demonstrated that remote monitoring adopting invasive measurements (e.g., pulmonary artery pressure sensors) reduced HF hospitalisations through pressure-guided therapy adjustment [37]. Although invasive and less scalable, this work provides proof-of-concept evidence that physiology-guided care can improve clinical outcomes in HF patients. In the past decade, a variety of wearable technologies have been proposed to non-invasively monitor HF, each with distinct strengths and limitations (Table 1). Consumer-grade wearables (e.g., smartwatches) cover a substantial part of the monitoring area, and are typically able to measure heart rate, heart rate variability (HRV), respiratory rate using photoplethysmography (PPG) and accelerometry [38, 39]. Consumer-grade wearables are scalable, affordable and are generally well-accepted by patients, enabling feasible, continuous longitudinal monitoring without medical stigma. In the early post-discharge phase, wearables seem particularly suited to capture trends in autonomic balance, circadian rhythm, physiological stress, and activity recovery [40]. Nonetheless, their role in guiding GDMT optimisation during early HF stabilisation remains exploratory as they do not provide a direct surrogate of hemodynamic status.

**Table 1:** Novel technologies that allow continuous assessment of haemodynamic surrogates of heart failure filling status

Technology	Parameters Measured	Strengths	Limitations	Clinical Maturity	Potential in Early Stabilisation
Smartwatches (PPG-based) [38]	HR, HRV, activity, RR	Scalable, high adherence, low cost	Indirect hemodynamics, motion artefacts	Validated for HR/arrhythmia	Moderate (requires algorithm integration)
Multi-sensor patches [41]	ECG, RR, activity, impedance	Higher signal fidelity, short-term continuous data	Limited wear-time, cost	ECG validated; impedance mixed	High (allows short-term post-discharge monitoring)
Thoracic impedance [42]	Fluid surrogate	Direct congestion proxy	Limited specificity	Mixed evidence	Moderate
Speech analysis [43]	Speech	Low cost, possibility for early prediction	Early stage validation	Experimental	Promising but immature
Circadian rhythm [40]	HR, circadian parameters	Not sensitive to moment-to-moment variation	Early validation stage, single sensor	Experimental	Promising but immature
Seismocardiography [44]	Mechanical cardiac vibrations	Potential haemodynamic insight	Early-stage validation	Moderate prediction performance	Promising but immature
Invasive pulmonary artery pressure sensors [25]	Filling pressures	Strongest haemodynamic signal; outcome evidence	Invasive, costly	Robust evidence	High but limited scalability

*ECG* electrocardiogram, *HR* heart rate, *HRV* heart rate variability, *PPG* photoplethysmography, *RR* R-R peak interval

Past years, several other alternatives have been introduced to non-invasively monitor patients with HF. A first alternative relates to adhesive multi-sensor patches, which may enable continuous ECG, respiratory rate, activity, and thoracic impedance. Adhesive patches offer superior signal fidelity compared to wrist-worn devices, though they are only practically feasible for a limited duration. Therefore, adhesive patches are particularly well-suited to the first few weeks post-discharge, when haemodynamic instability and GDMT up-titration are most dynamic [39]. A second alternative monitoring system, focused on long-term use, relates to thoracic impedance which can be measured using textile-based wearable sensors and reflects pulmonary fluid accumulation. Unfortunately, these monitors have modest stand-alone diagnostic performance due to false positives [42]. Third, a more recently introduced alternative to monitor patients refers to speech analysis. Speech analysis may detect early HF-related changes driven by pulmonary congestion, which alters mucosal conditions and vocal fold biomechanics [43]. This approach remains in early validation stages and requires future studies to understand its full potential, with privacy concerns being appropriately addressed. Finally, emerging monitoring technologies such as seismocardiography and ballistocardiography aim to non-invasively estimate stroke volume and mechanical cardiac function [44, 45], but have not been extensively validated in ADHF populations.

Collectively, improving early post-discharge stabilization requires a shift from episodic symptom assessment to continuous, physiology-informed risk management. Wearables may reduce clinical inertia and enable safe GDMT optimization. However, existing wearables require rigorous validation and outcome-driven evaluation to determine their true clinical potential. Within these developments, adopting a multi-modal parameter approach seems promising as it may mitigate limitations of individual sensors (e.g. noise) [41, 46]. The clinical value of these monitors may be further optimised when combined with minimally invasive approaches to assess biomarkers (e.g., NT-proBNP) [47]. Future studies in ADHF populations are therefore, required to identify optimal multiparameter models and settings, including their implementation, alignment with clinical workflows, and its cost-effectiveness.

## Low Functional Capacity: Role for Home-based Cardiac Rehabilitation

Exercise training is a class IA recommendation for patients with HF in international guidelines and constitutes a cornerstone of comprehensive HF management [32, 33, 48]. Recent meta-analyses demonstrated that participation in exercise-based cardiac rehabilitation (CR) leads to improvements in functional exercise capacity (+ 21.0 m; 95%CI: 1.6–40.4 m on 6-minute walking distance), clinically-relevant improvements in quality of life (5.7–7.4 point improvement on the Minnesota Living with Heart Failure questionnaire), and 31% lower risk of all-cause hospitalisations up to 12 months of follow-up [49, 50]. Beyond these clinical benefits, the reduced risk of hospital admission is relevant from a health-economic perspective as hospitalisation accounts for ~ 70% of HF-related healthcare costs (€25,500 per patient/annum [12, 51]).

Despite the well-established benefits, participation rate of HF patients in exercise-based CR remains low, with only 5–10% of eligible patients enrolling in CR [21, 22]. The reasons are complex and multifactorial [52–54]. One potential reason is that conventional CR is predominantly delivered using a “one-size-fits-all” approach, which insufficiently accounts for patient heterogeneity in HF populations. Older age, for example, has been associated with lower enrolment rates [52–54]. Indeed, a recent meta-analysis across 60 trials including 8,782 patients with HF reported a mean age of 63.3 years [50], whereas in routine practice the average age of patients with HF approaches 70 years. This is important, as both prevalence and hospitalisation risk increase markedly with advancing age [50, 55]. Furthermore, HF patients often suffer from comorbidities and frailty, with > 90% of hospitalised HF patients having > 1 comorbidity [56] and > 50% being considered frail [57]. Unfortunately, these vulnerable individuals with HF are frequently underrepresented in clinical practice, and typically excluded from clinical trials [58]. Collectively, these differences between trial and real-world HF populations underscore persistent knowledge gaps.

Knowledge gaps pertaining to the benefits of CR are particularly evident in the context of ADHF [12, 59]. During and following hospitalisation for acute decompensation, these patients commonly experience low physical capacity, impaired quality of life, and exhibit a markedly elevated risk of short-term hospitalisation [12, 60]. Observational studies suggest that early in-hospital rehabilitation is associated with improvements in physical function and reductions in rehospitalisation risk compared with standard HF management alone [28, 61, 62]. The pivotal REHAB-HF trial directly addressed this high-risk population through enrolling older patients recently hospitalised for ADHF (72.7 ± 8.1 years), most of whom were (pre-)frail (97%) and had multiple comorbidities (mean 5.2 ± 2.0) [63]. A multi-domain, tailored, center-based exercise intervention initiated during or shortly after hospitalisation resulted in greater improvements in physical function compared with usual care [63]. These findings demonstrate that individually tailored rehabilitation is feasible and effective even in highly vulnerable patients.

Despite these encouraging findings, important knowledge gaps remain. Most CR programs for patients with ADHF are delivered in-hospital, which creates barriers related to scheduling constraints and travel (because of time, costs, mobility limitations and/or logistical constraints) [52–54]. Indeed, ~ 30% of eligible patients for REHAB-HF did not participate due to logistical constraints [63]. In contrast, home-based CR, including tele-rehabilitation approaches, offers a promising strategy to overcome such barriers by reducing travel demands, increasing flexibility of scheduling/access, and facilitating individualised care [64, 65]. While evidence suggests that home-based and center-based CR achieve comparable outcomes in the HF population [64, 65], a tailored, co-created, home-based CR programme specifically designed for older and frail patients following hospitalisation for ADHF has not yet been established. Future research should therefore prioritise the development and evaluation of personalised home-based CR for these vulnerable patients.

Non-invasive remote monitoring may be an important source to further improve the efficacy of CR in patients with HF following acute decompensation. Firstly, artificial intelligence enables to continuously personalize exercise prescriptions (e.g. in volume, intensity, duration) based on individual physiological data, comorbidities, energy expenditure and training response. This may be relevant in HF patients, as many patients struggle with the high energy demands of their activities of daily living [66–68]. Artificial intelligence-supported home-based CR interventions may allow exercise load to be dynamically adjusted to the patient’s energy reserve. Secondly, consumer-grade wearables may increase overall adherence and compliance to CR through tracking physical activity targets using these devices [69]. Adherence to CR may be further improved by artificial intelligence delivering motivational prompts [68, 70]. Together, introduction of wearables and artificial intelligence-supported tools may enable personalization of CR delivery and improve adherence to CR, potentially resulting in improvement in functional capacity and quality of life of HF patients in this high-risk population.

## Challenges in Early Prediction of Re-admission: Data-driven Detection

Timely identification of temporal abnormalities in continuously-measured physiological signals might enable earlier clinical intervention and prevent clinical progression [71]. However, these large, multimodal datasets are difficult to interpret clinically [72, 73]. In fact, such datasets entail the risk of introducing a time-consuming care path with limited clinical value. This underscores the need for data sensemaking for interpretation of these vast datasets. From a technological and analytical perspective, this process of sensemaking involves transforming raw data into meaningful information to allow interpreting complex and abundant data [73, 74]. Sensemaking can be achieved by adding a human-computer interaction perspective, leading to improved usability, user experience, and workflow-aligned presentation. This allows patients and clinicians to interpret, trust and apply insights from these multimodal datasets [75]. By incorporating these multidisciplinary insights, data sensemaking strategies can provide oversight and meaning to available information and enable stakeholders to make better informed decisions [72, 76].

An aspect inherent to data sensemaking is machine learning (ML). ML is a form of artificial intelligence, consisting of algorithms and models that are not completely predefined but learn from data [72, 77, 78]. ML can be used to extract meaningful variables, identify data patterns linked with early deterioration of HF and integrate data from many rich and distinct data sources [72]. ML ranges in complexity from simple and explainable models (e.g. decision trees and regression) to highly-complex, black-box models (e.g. convolutional neural networks). These models typically leverage large datasets to identify complex relationships between data and outcomes. To accommodate data sensemaking, ML-based models may facilitate detection and prediction of early clinical deterioration in HF patients. For example, deep learning models were previously trained based on rich accelerometry data from consumer-grade wearables to derive one’s estimated peak oxygen consumption, which was strongly associated with incidence of HF-related hospitalisation [79]. Additionally, a ML model based on a multimodal system (i.e. ECG, accelerometry and skin impedance and temperature) successfully detected clinical worsening of heart failure with high sensitivity and specificity [80]. Together, these observations highlight the potential of ML to predict clinical worsening of HF.

An important and inherent challenge of ML is the identification of a set of clinically meaningful physiological variables. When used in a ML model, data collected via physiological sensors may be converted into metrics relevant to the outcome of interest through a series of advanced data transformations. Due to the complexity of these ML models, their output should be clinically interpretable and actionable, so that medical decision making is augmented beyond clinical judgment alone [81]. For example, if an ML model is trained on data sensitive to changes in HF status (e.g. pulmonary artery pressure, speech analyses), it may enable clinicians to identify patients at high risk of acute decompensation well before the patient becomes symptomatic [40]. This early warning could allow clinicians to timely adjust therapy or provide patient counselling, potentially attenuating or reversing clinical deterioration. Importantly, making use of continuous data for patient monitoring will only entail clinical benefits when embedded in a renewed HF care pathway. This renewed pathway should consist of clearly defined responsibilities for healthcare professionals, including guidelines on how to review, understand and act upon signals within predefined time windows.

## The Importance of Co-creation in Innovating Healthcare

Despite the rapid expansion of home-based monitoring and digital innovations, primarily accelerated during and following the COVID‑19 pandemic, the effects of telehealth and telecare interventions remain far from unequivocal [82]. Indeed, resistance to change may become a significant barrier to implementation [83]. Recent observations have shown that a top-down approach without proper involvement of stakeholders may fail to demonstrate clinical benefit [84]. Therefore, implementing telehealth and telecare interventions in clinical practice may benefit from early involvement of relevant stakeholders in the design process, so-called “co-creation”. Co-creation is a collaborative approach of creative problem solving engaging diverse stakeholders at all project stages, from determining and defining the problem through to the final stages of a project. Systematic reviews have demonstrated the benefits of co-creating interventions, with improved adherence to interventions and strong effects on complex health behaviors related to one’s lifestyle [85].

Previous experiences highlight that researchers, clinicians and other stakeholders should work iteratively to tailor innovations to real-world practice. Potential implementation challenges should be identified and addressed early in the design process, preferably prior to and during the clinical research. This approach bridges the gap between designers, engineers and end-users, thereby facilitating an effective clinical decision support system. Design priorities tend to be shaped by clinical, operational, or cost considerations rather than by patient-centered desires and requirements [86–90]. Similarly, limited involvement of clinicians in design and implementation introduces the risk of clinicians resisting the implementation of (technological) innovations from a fear of workflow disruption or lack of usefulness [82].

**Fig. 3:**
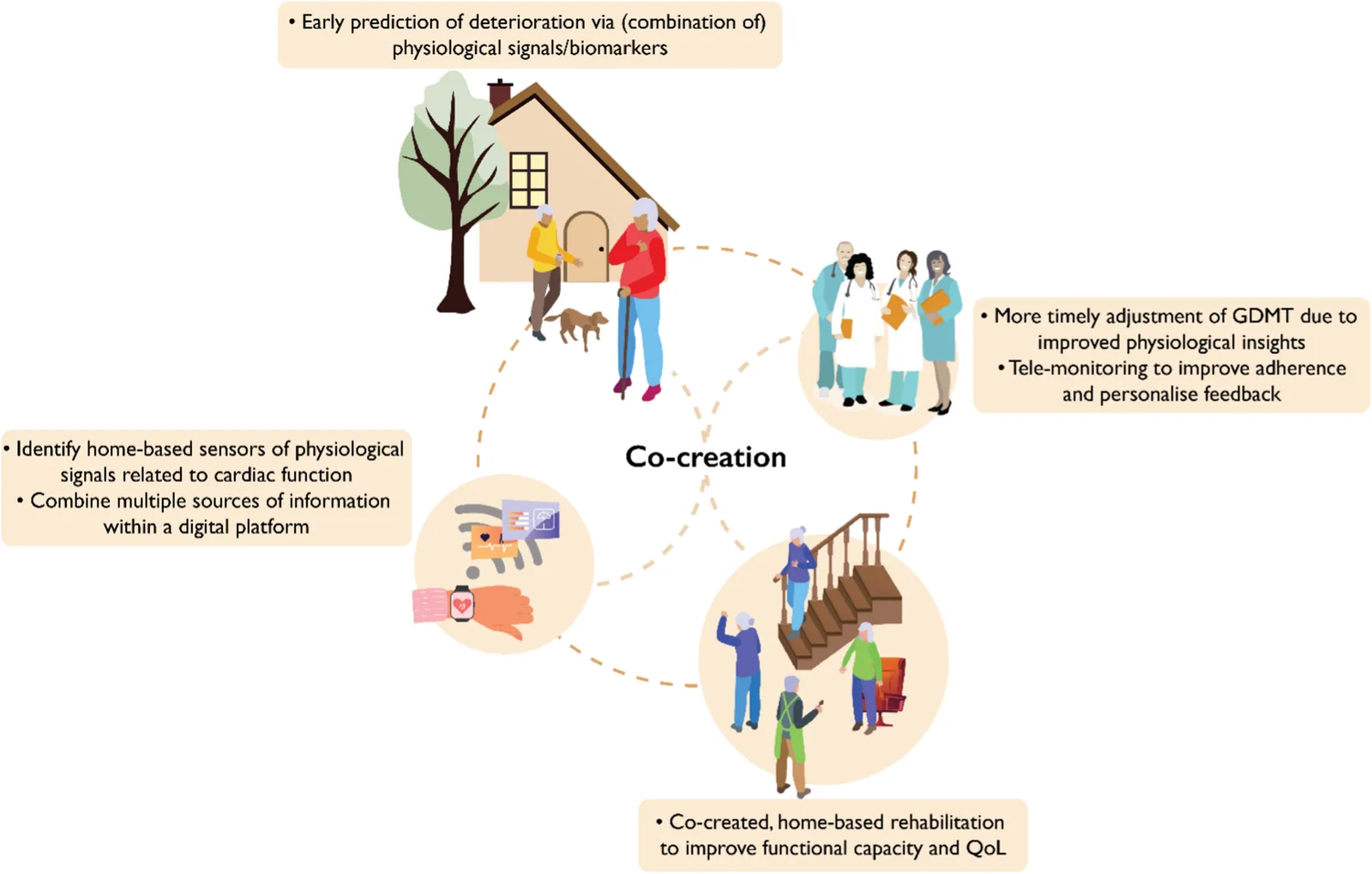
Future role of non-invasive, home-based monitoring and rehabilitation for patients with heart failure following decompensation. *Non-invasive*,* physiological signals should be identified that are related to cardiac function*,* with information from multiple sources being integrated in a user-friendly and inclusive digital platform. Using these physiological signals may enable prediction of early deterioration in the home environment. Subsequently*,* this information may enable more timely-adjustment of GDMT and improve adherence. Additionally*,* home-based cardiac rehabilitation may improve functional capacity and clinical outcomes. Collectively*,* such approaches should be co-created*,* continuously tailoring technological innovations to the needs*,* requirements and priorities of all relevant stakeholders. GDMT guideline-directed medical therapy*,* QoL quality of life*

To ensure that home-based, telehealth interventions are effective, context-sensitive and sustainable, it is essential to identify and actively engage a wide array of potential stakeholders, including patients, caregivers, healthcare professionals, and policy-makers (Fig. 3). These stakeholders should be included continuously throughout the problem formulation, design and implementation. Moreover, in selecting relevant stakeholders, it is imperative to be diverse in terms of age, ethnic background, level of education, level of literacy, health status (e.g. comorbidity and type of HF), and social network/embeddedness. This diversity ensures input from a wide range of experiences and practical realities. Adopting these key principles lead to interventions being tailored towards the needs of the stakeholders and, consequently, increase chances for successful implementation of technology in (clinical) practice [86, 91, 92]. In this approach, it is essential to continually assess and bring into conversation the needs, requirements, and priorities of all stakeholders. Through this iterative approach, the stakeholders can continually shape the innovation under design for a robust fit with what is needed and what will work across diverse real-world settings.

## Implementation of Innovations in Healthcare: the Role of Health Technology Assessment

Healthcare systems are met by a virtually unlimited demand to provide care despite continuously being challenged by limited (financial/staff) resources. Therefore, a systematic evaluation of the expected benefits, costs and required resources should be made at the patient-, healthcare organisation, and societal level to determine the expected impact of implementing and reimbursing innovations in healthcare. This multidisciplinary, systematic evaluation is typically referred to as Health Technology Assessment (HTA). Insights from HTA analyses provide tangible information for policy- and decision making to facilitate an equitable, efficient and high-quality healthcare system. To provide a comprehensive oversight of the impact of an innovation, HTA should capture all relevant consequences, that is, both at the short term but also at the intermediate and long-term. This typically means that long-term outcomes must be estimated using simulation modelling that integrates all available evidence, and extrapolates effects based on evidence or assumptions across the entire patient lifetime [93]. Historically, HTA was typically applied only after implementation of an innovation in practice. However, over the last two decades, initiatives have increasingly emerged to perform HTA already early on, during the design and development process, to increase the potential value of innovations, and thereby their change of successful implementation (so-called ‘early-HTA’) [94, 95].

When contextualized to HF care, HTA is associated with some specific HF-related challenges. For example, patients with HF show high within-and between-patient heterogeneity in treatment responses, which markedly influences the disease course and outcomes. Accounting for this heterogeneity requires development and validation of a simulation model at the individual-level, such as a discrete event simulation that models the care pathway as a series of events (e.g. hospitalisation event) that occur over time and depend on patient characteristics, which themselves can also change over time (e.g. HF-related risk factors) [96]. A second challenge adopting HTA for the evaluation of (technological) innovation for ADHF patients relates to behaviour-driven effectiveness. For example, benefits of cardiac rehabilitation largely depend on patient motivation and adherence to cardiac rehabilitation programmes. Understanding determinants of non-completion and patient motivation is therefore needed as a foundation for accurate simulation modelling across time. A third challenge relates to changes in health system workflow. For example, implementation of telemonitoring and home-based cardiac rehabilitation relocates care at the hospital to care at home. This inherently affects clinical workflows of healthcare professionals (e.g. in volume, intensity and content), and should be incorporated in HTA models to ensure inferences are both realistic and valuable. Finally, one should consider that impact of the (technological) innovation will differ across patient subgroups (e.g., age, sex, and frailty). Identifying in which subgroups, or at which threshold for readmission risk [97], the innovation is (cost-) effective can guide its implementation and support reimbursement decisions.

### Limitations of this Review

A limitation of this review is its narrative nature rather than adopting a pre-defined search strategy as in systematic reviews.

## Conclusions

An important challenge in the management of HF remains acute decompensation, especially in relation to the poor post-hospital recovery. During this post-hospitalisation phase, infrequent and one-size-fits-all surveillance contributes to poor patient management and suboptimal treatment. Furthermore, HF patients demonstrate low levels of functional capacity, and remain at increased risk for readmission due to sudden deterioration. Interestingly, in the past decade, technological innovation allowed for improvement in non-invasive monitoring of relevant physiological signals, but also home-based delivery of cardiac rehabilitation. To translate these advances into effective real-world care, we advocate adopting an inclusive (e.g., age, sex, multimorbidity, ethnicity, education level) and multidisciplinary (e.g., cardiology, technology development, data analytics, design) co-creation approach. This co-creation process is characterised by actively engaging stakeholders (e.g., patients, caregivers, healthcare professionals, data scientists, policy-makers, insurance companies) in design, planning and implementation of research. Such approach ensures that innovations are context-sensitive, effective and sustainable in the ‘real-world’. To facilitate implementation of innovations, we strongly support the use of HTA techniques. Based on current technological developments, we propose the following fields of innovation to improve healthcare and (clinical) outcomes for patients with ADHF:

1. *Rapid stabilisation after ADHF* → Driven by recent developments, we propose future studies to evaluate the feasibility and effectiveness of home-based, non-invasive monitoring of relevant physiological signals to improve and accelerate post-discharge stabilisation. These steps may require validation of innovative technology against gold standard technology. This would allow for regular, valid and feasible evaluation of relevant (combinations of) physiological signals. Combined with innovations in minimally invasive biomarker assessment, these steps may allow for earlier detection of clinical deterioration and timely optimisation of GDMT. Ultimately, the goal is clinically actionable monitoring that reduces treatment variability, mitigates clinical inertia, and accelerates safe stabilisation in the early post-discharge period.
2. *Improvement of functional capacity* → Previous observations reinforce the clinical benefits of cardiac rehabilitation, with comparable patient-outcomes following hospital- and home-based cardiac rehabilitation. Following co-creation principles, and acknowledging the barriers for HF patients following acute decompensation, we recommend future studies to co-design and evaluate an inclusive, transmural home-based integrated cardiac rehabilitation programme. We highlight the importance of optimization of adherence to lifestyle changes, as higher adherence rates are associated with lower risks for mortality and morbidity [98]. By adopting user-friendly and inclusive eHealth platforms, one should consider aligning content to personal needs and to follow behaviour change theory [99].
3. *Early prediction clinical deterioration* → Previous studies provided support for the concept that changes in physiological parameters precede noticeable symptoms and clinical deterioration in patients with HF. We recommend future studies to examine the validity and feasibility of non-invasive monitors as an early warning to predict clinical deterioration. These monitors likely lead to (a combination of) physiological signals, including (but not limited to) vascular or cardiac hemodynamics, neurohormonal status and filling pressures. An associated challenge is that data collection, use and storage are user-friendly (for patient, caregivers and healthcare professionals) and aligns with (inter)national regulations on data safety.

Collectively, these innovations may facilitate more efficient patient management systems, improve recovery and guidance following episodes of decompensation, and ultimately enhance clinical outcomes and quality of life in patients with HF.

## Key References

- Brugts JJ, Radhoe SP, Clephas PRD, Aydin D, van Gent MWF, Szymanski MK, et al. Remote haemodynamic monitoring of pulmonary artery pressures in patients with chronic heart failure (MONITOR-HF): a randomised clinical trial. Lancet. 2023;401(10394):2113-23.
  - ○ The paper demonstrated that remote monitoring of invasive measurements reduced HF rehospitalisations through pressure-guided therapy adjustments, providing proof-of-concept evidence that physiology-guided care can improve clinical outcomes in HF patients.
- Scholte NTB, van Ravensberg AE, Shakoor A, Boersma E, Ronner E, de Boer RA, et al. A scoping review on advancements in noninvasive wearable technology for heart failure management. NPJ Digit Med. 2024;7(1):279.
  - ○ The paper outlines advancements in non-invasive wearable technology for HF management.
- Molloy CD, Long LD, Mordi IR, Bridges C, Sagar VA, Davies EJ, et al. Exercise-based cardiac rehabilitation for adults with heart failure-2023 Cochrane systematic review and meta-analysis. European Journal of Heart Failure. 2023;25(12):2263-73.
  - ○ Cochrane review on the effects of CR in HF, which documents its strong clinical benefits.
- Kitzman DW, Whellan DJ, Duncan P, Pastva AM, Mentz RJ, Reeves GR, et al. Physical Rehabilitation for Older Patients Hospitalized for Heart Failure. New England Journal of Medicine. 2021;385(3):203-16.
  - ○ A randomized trial (REHAB-HF) highlighted the feasibility and benefits of a multi-domain, individually-tailored exercise intervention ininiated during or shortly after hospitalisation in patients with increased age, frailty and multimorbidity.

## Data Availability

No datasets were generated or analysed during the current study.
